# VisionMetric Suite for periocular measurement in ophthalmic plastic surgery: a preliminary single-center validation study

**DOI:** 10.3389/fmed.2026.1855884

**Published:** 2026-06-15

**Authors:** Tengfei Wang, Yunke Li, Kun Wang, Lingyi Zhang, Yingying Tian, Mengru Pang

**Affiliations:** 1 Department of Burns and Plastic Surgery, The Affiliated Hospital of Guizhou Medical University, Guiyang, China; 2 Department of Dermatology, The Second People's Hospital of Guiyang, Guiyang, China

**Keywords:** artificial intelligence, automated assessment, facial landmark detection, image analysis, ophthalmic plastic surgery, periocular measurement

## Abstract

**Background/objectives:**

Periocular plastic surgery lacks standardized, objective tools for preoperative planning and postoperative evaluation, currently relying on subjective assessment and manual measurements. This study aims to validate VisionMetric Suite, an artificial intelligence-based automated periocular measurement tool, and compare its performance with manual methods.

**Methods:**

We developed VisionMetric Suite based on the dlib library and shape estimation algorithms. The tool automatically measures 60 quantitative periocular parameters. The software was evaluated on 226 frontal facial photographs for recognition rate, processing speed, and user satisfaction. Thirty images were randomly selected to assess the agreement between automated and manual measurements and the repeatability of the AI tool using ICC and Bland-Altman analysis. Clinical utility was demonstrated through a case of left congenital ptosis.

**Results:**

The software achieved a recognition rate of 94.7% and an average processing time of 2.8 ± 0.4 s per image. User satisfaction among 10 physicians averaged 4.3/5.0. Agreement between automated and manual measurements showed a mean ICC of 0.94 (95% CI: 0.91–0.97), and repeatability of repeated AI measurements showed a mean ICC of 0.96 (95% CI: 0.92–0.98). Bland–Altman analysis confirmed these findings, and a representative clinical case illustrated the software's practical utility.

**Conclusions:**

This preliminary single-center validation study suggests that VisionMetric Suite can serve as a rapid, objective periocular measurement tool with good agreement compared to manual methods, representing an initial step toward automated assessment in ophthalmic plastic surgery.

## 1 Introduction

Periocular surgery including blepharoplasty, brow lift, ptosis correction, and periorbital deformity repair is one of the most frequently performed procedures in aesthetic plastic surgery worldwide (1). Accurate preoperative planning and objective postoperative evaluation are essential for optimizing surgical outcomes and ensuring patient satisfaction. However, current clinical practice still relies heavily on subjective observation and manual measurements using rulers or calipers (2, 3).

Manual measurements have well documented limitations. They are time-consuming (4), operator-dependent, and exhibit significant interobserver and intraobserver variability (5). While three-dimensional imaging systems offer high precision, they are expensive and impractical for routine use (6, 7). Software tools such as ImageJ have been used to measure ocular parameters; however, their reliability varies with user expertise, and the process remains labor-intensive (8, 9).

Recent advances in machine learning-based facial landmark detection offer new possibilities for automated assessment (10, 11). However, existing AI-based periocular tools have limitations. FACE-gram and Emotrics were primarily designed for facial palsy evaluation rather than periocular plastic surgery (12, 13). Other reported tools focus on a limited set of parameters such as MRD1 and palpebral fissure height (14, 15) or require specialized equipment (16). None of the available tools simultaneously provide comprehensive, rapid, and fully automated measurement of eyelid morphology, brow morphology, and brow eye relationships.

Therefore, a clear unmet clinical need exists for a standardized, objective, and efficient periocular measurement tool that can be easily integrated into routine clinical workflow for preoperative planning and postoperative assessment.

To address this gap, we developed VisionMetric Suite, an intelligent software tool based on the dlib library and shape estimation algorithms. The tool detects 84 facial landmarks including 38 periocular specific points and generates 60 quantitative parameters within 3 s. This study presents a preliminary single center validation of the tool's performance, assessing its agreement with manual measurements, repeatability, recognition rate, and clinical usability.

## 2 Materials and methods

### 2.1 Study design and ethics

This prospective observational study was approved by the Ethics Committee of Guizhou Hospital of the First Affiliated Hospital, Sun Yat-sen University (Approval No.: GMCAH-GA20250923) and followed the Declaration of Helsinki. Written informed consent was obtained from all participants. Clinical trial registration: not applicable.

### 2.2 Image acquisition

Standardized frontal digital photographs were taken using a digital camera (Canon 1500D, Canon Corporation, Tokyo, Japan) with a 24–120 mm lens. During photo sessions, patients were seated 1.0 m from the camera, maintaining a natural head position with eyes open and frontalis muscles fully relaxed. All images were taken under standardized lighting conditions.

#### 2.2.1 Inclusion and exclusion criteria

The 226 images, including normal faces, preoperative and postoperative blepharoplasty, ptosis, and dermatochalasis cases, were selected from patient records of our department between 2023 and 2025 according to predefined criteria. Inclusion criteria were: (i) frontal view with both eyes visible; (ii) neutral expression with eyes open and frontalis muscle relaxed; (iii) clear image with uniform illumination and no motion blur; (iv) clear visualization of periocular structures (eyebrows, eyelids, medial canthi, lateral canthi). Exclusion criteria were: (i) head rotation >10°; (ii) presence of glasses, heavy makeup, or periocular obstruction; (iii) exaggerated facial expressions; (iv) uneven illumination, low resolution, or motion artifacts. All images meeting these criteria were included without further selection to avoid bias.

### 2.3 AI tool measurements (VisionMetric Suite)

#### 2.3.1 Landmark detection model

VisionMetric Suite is built on the dlib library's pre-trained 68-point facial landmark detection model (17). To enhance periocular analysis, we trained a shape estimation model using the Ensemble of Regression Trees algorithm to predict 16 additional periocular-specific landmarks (eight per side). The model was trained on the iBUG 300-W dataset (https://ibug.doc.ic.ac.uk/resources/300-W/), which contains over 2,400 manually annotated facial images with 68 landmarks per image, using an 80/10/10 split (training/validation/test).

##### 2.3.1.1 Annotation of additional landmarks

**Figure 1:**
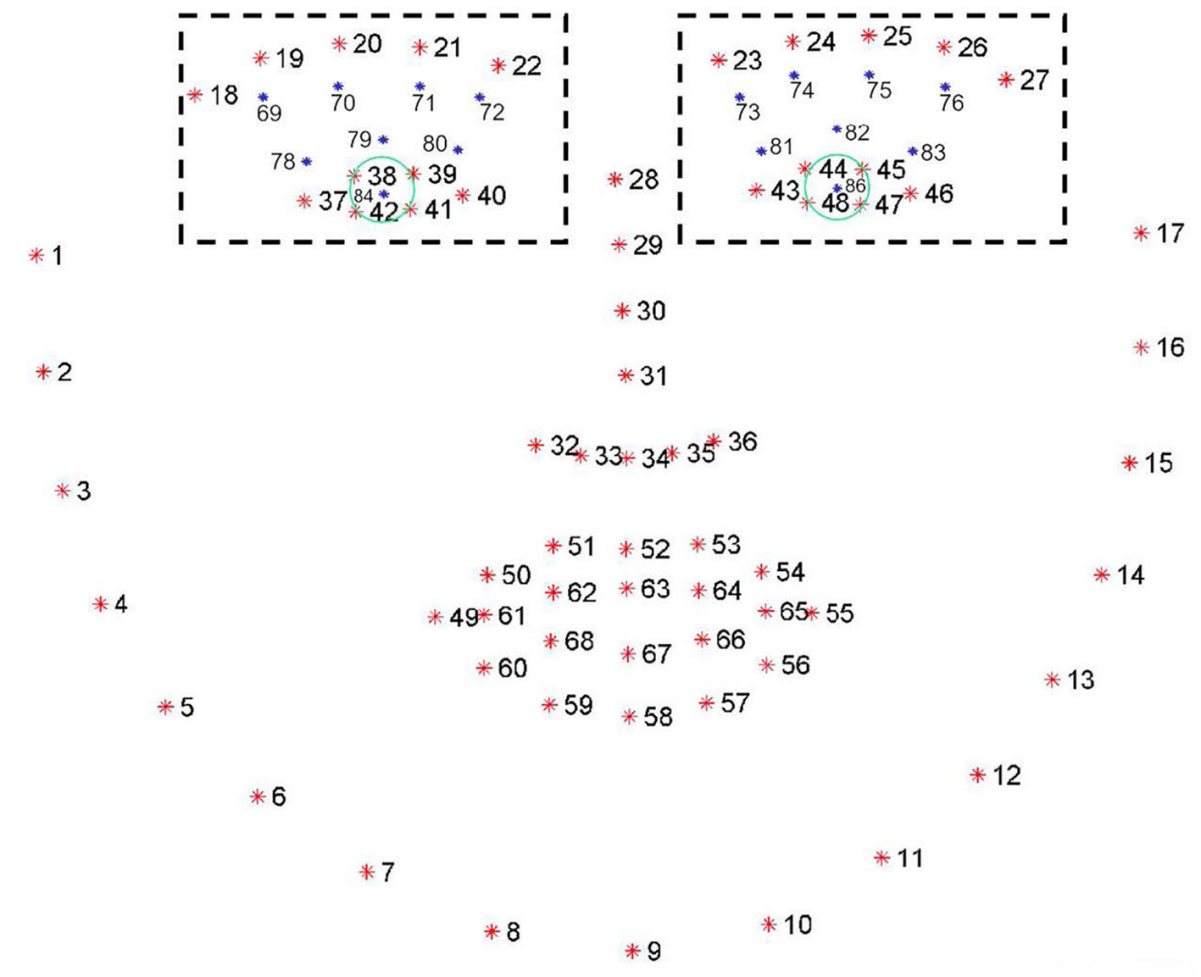
Distribution of the 84 facial landmarks detected by VisionMetric Suite. Red points represent the original 68 facial landmarks from the dlib library, including the contours of the jaw, eyebrows, nose, eyes, and mouth. Dark blue points indicate the 16 newly added periocular-specific landmarks (eight per side), comprising four inferior brow margin points, three supratarsal fold points, and the pupillary center for each eye. The black dashed box highlights the periocular region, where a total of 38 landmarks (22 from the original 68-point set and 16 newly added points) are concentrated. This combined landmark set serves as the basis for computing the 60 quantitative periocular parameters.

To obtain ground-truth labels for the 16 additional landmarks, we randomly selected 200 images from the same dataset. These images were manually annotated by two experienced ophthalmic plastic surgeons (TW and MP, each with >5 years of clinical experience), who were not involved in the model development to avoid bias. The 16 additional landmarks per image included: four inferior brow margin points, three supratarsal fold points, and the pupillary center (shown as dark blue points in Figure 1). The two annotators worked independently and were blinded to each other's annotations. Annotation consistency was assessed using the intraclass correlation coefficient (ICC), yielding a value of 0.96 (95% CI: 0.94–0.98), indicating excellent agreement.

##### 2.3.1.2 Model training and testing for the 16 additional landmarks

Based on these 200 annotated images, we established a rigorous evaluation protocol. The 200 annotated images were split into 140 for training, 30 for validation, and 30 for independent testing (70/15/15). The shape estimation model was trained on the training set (*n* = 140), optimized on the validation set (*n* = 30), and evaluated on the independent test set (*n* = 30). On this test set, the model achieved a Normalized Mean Error of 0.042 ± 0.008 and a Success Detection Rate of 92.6%, confirming high prediction accuracy.

We adopted a two-stage cascaded framework: Stage 1 detects 68 base facial landmarks using dlib's pre-trained shape predictor; Stage 2 predicts 16 additional periocular points based on the 68-point output. For each eye side, the model predicts four inferior brow margin points, three supratarsal fold points, and the pupillary center. The same prediction rules were applied independently to the contralateral eye, yielding a total of 84 facial landmarks with 38 points concentrated in the periocular region (Figure 1).

#### 2.3.2 Calibration

To convert pixel distances to physical measurements, the software uses the mean horizontal iris diameter (11.77 mm) as an internal scale reference. The horizontal iris diameter was chosen as the calibration standard because it remains relatively constant across different gaze directions and its boundaries are clearly detectable. This value has been validated in human iris studies (18) and established as a calibration benchmark in automated facial measurement tools (12, 13). The algorithm automatically detects the limbus boundaries and calculates the iris diameter in pixels, establishing a pixel-to-millimeter conversion factor for each image.

#### 2.3.3 Automated parameter calculation

Following landmark detection and calibration, the software computes 60 quantitative parameters (Supplementary Table S1) across four major categories.

**Eyelid morphology:** lateral, central, and medial palpebral fissure height, palpebral fissure width, supratarsal fold height, MRD1 and MRD2, medial canthal angle, lateral canthal angle, corneal exposure area.

**Brow morphology:** brow height, brow length, brow peak position, brow curvature, brow area.

**Brow-eye relationships:** lateral, central, and medial brow-eye distance, brow-eye tilt angle.

**Interocular relationships:** intercanthal distance, interpupillary distance, bi-canthal distance.

#### 2.3.4 Workflow

**Figure 2:**
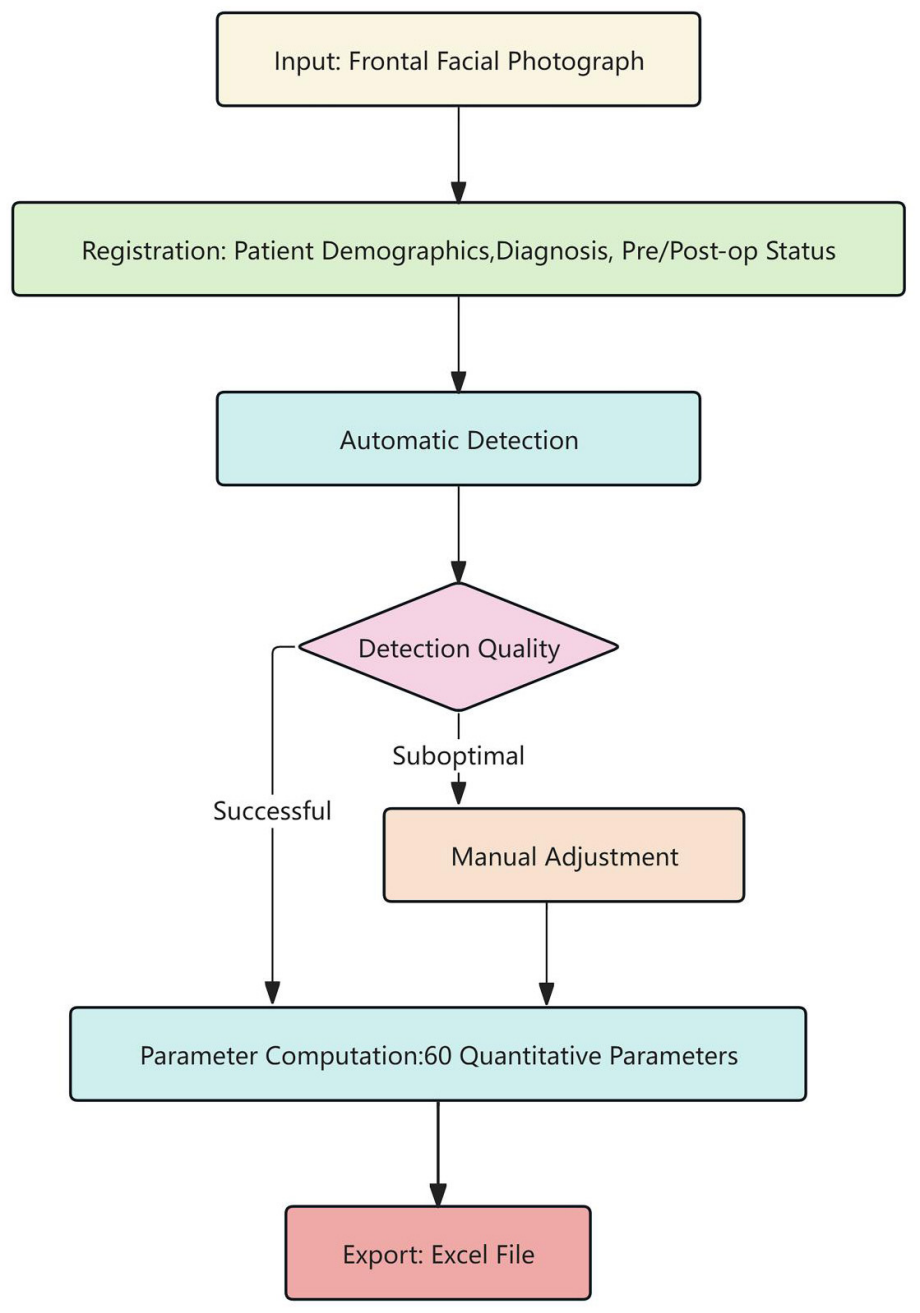
Workflow diagram of VisionMetric Suite.

The software workflow comprises six steps (Figure 2): (1) input of standard frontal facial photographs (JPEG/PNG) with neutral expression and unobstructed facial region; (2) registration of patient demographics, diagnosis, and preoperative/postoperative status; (3) automatic detection of 84 landmarks and 38 periocular-specific points, if any landmark is inaccurately placed, users can click and drag it to the correct anatomical position based on visual inspection (19, 20); (4) iris diameter detection and scale calculation; (5) computation of 60 quantitative parameters; and (6) export of measurement data as Excel files.

A video demonstration of the complete software workflow, from image input to data export, is provided as Supplementary Video S1.

#### 2.3.5 Software evaluation

To assess recognition efficacy and processing time, 226 frontal facial photographs were analyzed using VisionMetric Suite on a standard desktop computer with an Intel i5 processor and 8 GB RAM.

To assess clinical usability and acceptance, we invited 10 physicians familiar with periocular surgery to participate in a user satisfaction survey. The participating physicians represented different clinical experience levels: three attending physicians (>10 years of experience), three senior residents (3–10 years), and four junior residents (1–3 years). All participants had experience with manual caliper measurements but no prior exposure to automated measurement tools. Each physician independently analyzed 10 standardized periocular photographs using the software and then completed a structured questionnaire evaluating six dimensions of software performance on a 5-point Likert scale (1 = strongly disagree, 5 = strongly agree): ease of use, accuracy, time efficiency, clinical utility, reliability, and overall satisfaction.

### 2.4 Comparative analysis of AI and manual measurements

To evaluate the reliability of VisionMetric Suite, 30 images were randomly selected from the successfully recognized dataset. Seven key parameters were measured by the AI tool, including palpebral fissure height, MRD1, brow height, brow-eye distance, medial canthal angle, lateral canthal angle, and brow curvature. As a control, manual measurements were performed using ImageJ (version 1.46, National Institutes of Health, Bethesda, MD, USA) by two experienced surgeons. In cases of substantial disagreement, a third surgeon performed repeated measurements and adjudication. All manual measurements were performed in duplicate, and the average values were used for analysis. To ensure comparability, the same calibration value (11.77 mm) was used for both methods.

The agreement between automated and manual measurements (AAM) was assessed by comparing the AI tool's measurements against the average of the two surgeons' manual measurements. To further assess the repeatability of the AI tool (RAI), two attending physicians (both with >5 years of clinical experience) independently operated VisionMetric Suite on the same 30 images. During this analysis, operators were allowed to manually adjust landmarks based on the software's actual recognition performance on each image. The two physicians were blinded to each other's results. This experiment was designed to evaluate the stability of the AI tool across different users, reflecting real-world clinical scenarios where multiple clinicians may use the software. Both AAM and RAI were quantified using the intraclass correlation coefficient (ICC) with a two-way random effects model (ICC 2,1). Additionally, Bland–Altman analysis was performed to further assess agreement.

### 2.5 Clinical application illustration

To illustrate the practical application of the software, a patient with left congenital ptosis is presented as an example. The patient was an 18-year-old female with clinical findings of a markedly smaller left palpebral fissure compared to the right, elevated left brow position, and significantly increased left brow-eye distance. The patient underwent left levator aponeurosis shortening. Standard frontal photographs were obtained preoperatively and immediately postoperatively and analyzed using VisionMetric Suite.

### 2.6 Statistical analysis

All data were analyzed using SPSS version 26 (IBM Corporation, Armonk, NY, USA) and *R* version 4.3.3 (*R* Foundation for Statistical Computing, Vienna, Austria). User satisfaction scores were summarized using descriptive statistics (mean ± SD, range, and percentage of scores ≥4). Similarly, descriptive statistics were applied to the software recognition rate, image processing speed, and the time required for the 10 users to learn the software. Differences in overall satisfaction across physician experience levels were compared using one-way ANOVA. The agreement between AI tool measurements and manual ImageJ measurements was assessed using the ICC and Bland–Altman analysis.

## 3 Results

### 3.1 Software performance

VisionMetric Suite successfully recognized 214 of 226 frontal facial photographs, achieving a recognition rate of 94.7% (95% CI: 91.2%−97.1%). Recognition failure was defined as the inability of the software to automatically detect all 84 facial landmarks. Demographic analysis of the 226 images and detailed categorization of the 5.3% recognition failure rate are provided in Supplementary Tables S2, S3, respectively. Representative examples of recognition failure cases are shown in Supplementary Figure S1. Figure 1 shows the 84 detected landmarks (including 38 periocular-specific points) and the 60 periocular parameters generated. The average processing time per image was 2.8 ± 0.4 s.

### 3.2 User satisfaction

The overall satisfaction score averaged 4.3/5.0 (SD ± 0.4, 95% CI: 4.0–4.6). Dimension-specific scores are presented in Table 1. All physicians found the software intuitive (4.4 ± 0.4) and 90% agreed it required minimal training (4.3 ± 0.5). For accuracy, 80% confirmed good alignment with clinical judgment (4.1 ± 0.5) and 70% accurate landmark identification (4.0 ± 0.6). Time efficiency received the highest ratings (4.6 ± 0.3 and 4.5 ± 0.4). All physicians agreed on the clinical value for preoperative and postoperative assessment (4.3 ± 0.4 and 4.4 ± 0.4). Regarding reliability, 90% and 80% agreed on result consistency (4.2 ± 0.5) and stability across images (4.1 ± 0.5). Overall satisfaction was high (4.3 ± 0.4), with 90% willing to recommend the software (4.4 ± 0.5). All 10 physicians mastered basic operations within 15 min (mean: 11 min). Regarding willingness to incorporate the software into clinical workflow, the majority of physicians responded favorably. Satisfaction scores were generally consistent across the three experience levels: attending physicians (*n* = 3) gave a mean score of 4.4, senior residents (*n* = 3) gave 4.2, and junior residents (*n* = 4) gave 4.3. Due to the small sample size (*n* = 10), these findings should be interpreted as preliminary usability observations that warrant confirmation in larger, multi-center surveys.

**Table 1:** User satisfaction scores for VisionMetric Suite (*n* = 10 physicians).

Dimension	Assessment item	Mean score (SD)	Range	Score ≥4 *n* (%)
Ease of use	Software interface is intuitive and easy to operate	4.4 (0.4)	4–5	10 (100%)
	Can be used proficiently without extensive training	4.3 (0.5)	3–5	9 (90%)
Accuracy	Automated measurements highly consistent with clinical judgment	4.1 (0.5)	3–5	8 (80%)
	Software accurately identifies key periocular structures	4.0 (0.6)	3–5	7 (70%)
Time efficiency	Software significantly reduces assessment time	4.6 (0.3)	4–5	10 (100%)
	Improves work efficiency compared to manual measurement	4.5 (0.4)	4–5	10 (100%)
Clinical utility	Quantitative parameters valuable for preoperative planning	4.3 (0.4)	4–5	10 (100%)
	Quantitative parameters valuable for postoperative evaluation	4.4 (0.4)	4–5	10 (100%)
Reliability	Repeated analysis of same image yields consistent results	4.2 (0.5)	3–5	9 (90%)
	Measurement results stable and reliable across images	4.1 (0.5)	3–5	8 (80%)
Overall satisfaction	Overall satisfied with the software	4.3 (0.4)	4–5	10 (100%)
	Would recommend the software to colleagues	4.4 (0.5)	3–5	9 (90%)
Average total	Mean of six dimensions	4.3 (0.4)	3–5	-

SD, standard deviation; scoring used a 5-point Likert scale (1 = strongly disagree, 5 = strongly agree).

### 3.3 Agreement and repeatability of VisionMetric Suite

**Figure 3:**
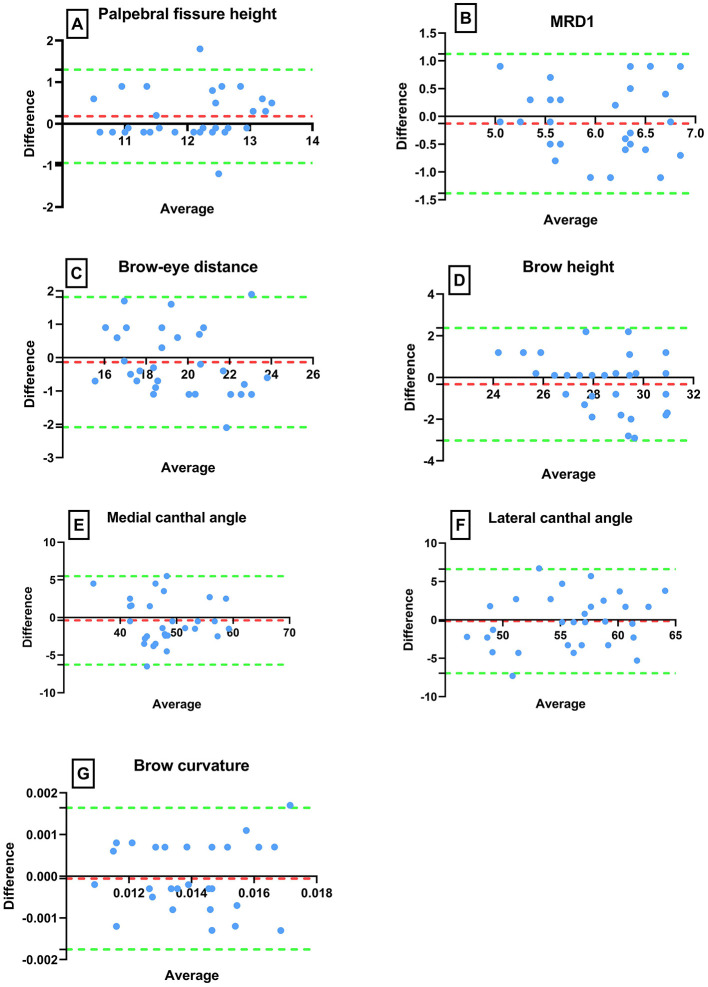
Bland–Altman plots for agreement between AI-based and manual measurements. Plots show the difference between AI and manual measurements (*y*-axis) against their mean (*x*-axis) for **(A)** palpebral fissure height, **(B)** MRD1, **(C)** brow-eye distance, **(D)** brow height, **(E)** medial canthal angle, **(F)** lateral canthal angle, and **(G)** brow curvature (*n* = 30). The solid red line represents the mean difference (bias), and the dashed green lines represent the 95% limits of agreement (bias ± 1.96 × SD). The shaded area indicates the 95% confidence interval of the limits of agreement.

**Figure 4:**
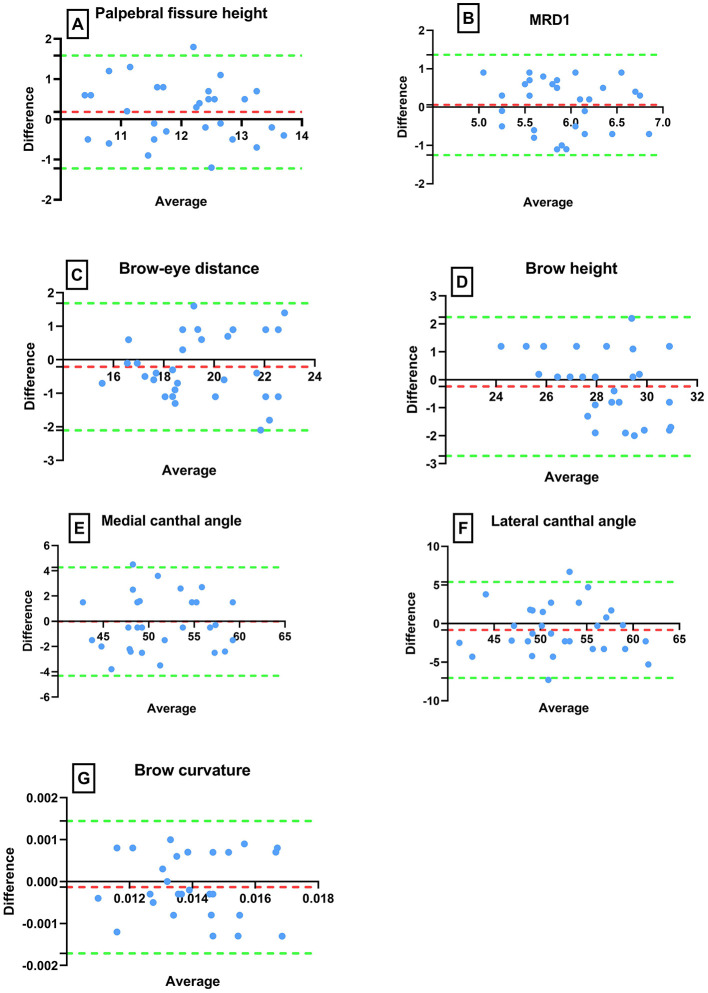
Bland–Altman plots for repeatability of the AI tool (RAI). Plots show the difference between two repeated AI measurements (*y*-axis) against their mean (*x*-axis) for **(A)** palpebral fissure height, **(B)** MRD1, **(C)** brow-eye distance, **(D)** brow height, **(E)** medial canthal angle, **(F)** lateral canthal angle, and **(G)** brow curvature (*n* = 30). The solid red line represents the mean difference (bias), and the dashed green lines represent the 95% limits of agreement (bias ± 1.96 × SD). The narrow limits of agreement confirm the excellent repeatability of the AI tool.

As shown in Table 2, VisionMetric Suite demonstrated excellent agreement between AAM across all parameters, with ICC values ranging from 0.87 to 0.98 and a mean ICC of 0.94 (95% CI: 0.91–0.97). RAI was also excellent, with ICC values ranging from 0.92 to 0.99. Notably, the repeatability ICC values were slightly higher than the agreement ICC values for most parameters, indicating the stability of the software across different users. Bland–Altman analysis further supported these findings. For the comparison between AI-based and manual measurements (Figure 3), the mean differences (bias) were close to zero for all parameters and the 95% limits of agreement (LoA) were narrow, indicating no systematic bias. For the repeatability of the AI tool (Figure 4), even smaller biases and narrower LoA were observed, confirming the exceptional consistency of the AI tool when applied repeatedly to the same images. No proportional bias was observed across the measurement range for any parameter.

**Table 2:** Agreement and repeatability of VisionMetric Suite measurements (*N* = 30).

Parameter	AAM ICC (95% CI)	RAI ICC (95% CI)
Palpebral fissure height	0.97 (0.94–0.99)	0.99 (0.98–1.00)
MRD1	0.96 (0.93–0.98)	0.98 (0.96–0.99)
Brow-eye distance	0.95 (0.91–0.98)	0.97 (0.94–0.99)
Brow height	0.94 (0.90–0.97)	0.96 (0.93–0.98)
Medial canthal angle	0.95 (0.92–0.98)	0.95 (0.91–0.98)
Lateral canthal angle	0.96 (0.93–0.98)	0.96 (0.93–0.98)
Brow curvature	0.87 (0.80–0.93)	0.92 (0.87–0.96)
Mean	0.94 (0.91–0.97)	0.96 (0.92–0.98)

ICC values >0.90 indicate excellent agreement; values between 0.80 and 0.90 indicate good agreement.

### 3.4 Clinical application illustration

**Figure 5:**
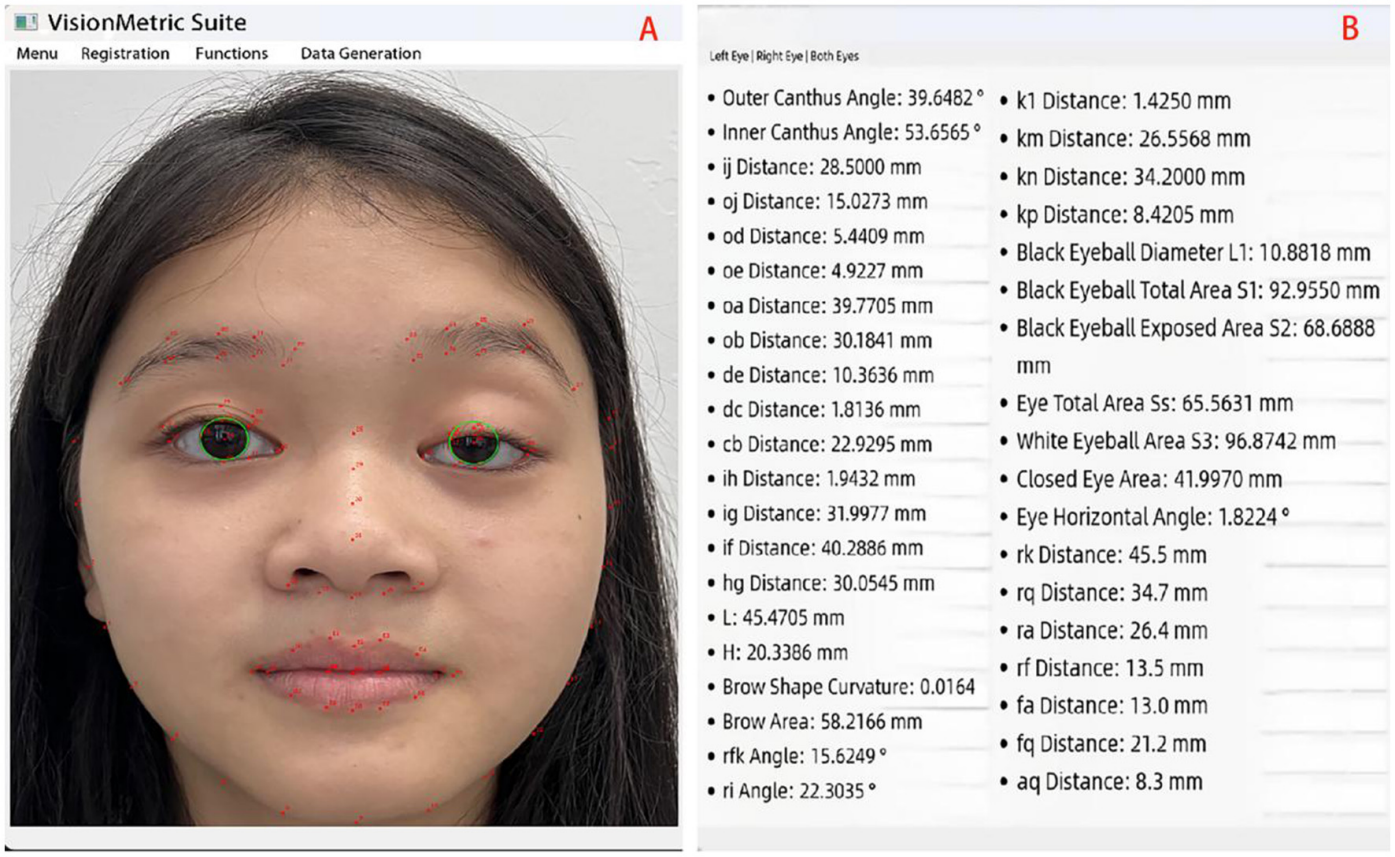
Software interface demonstration of the clinical example case. **(A)** Software interface showing 84 facial landmarks detected on a patient with left congenital ptosis. **(B)** Partial periocular measurement outputs displayed in the software interface.

To demonstrate the measurement output of VisionMetric Suite on actual images, a case of left congenital ptosis is presented as a usage example. This example is intended solely to demonstrate the software's output and potential for quantifying surgical changes, not as formal validation of the software as an outcome metric. Information on the example image is described in Section 2.5. The measurement outputs for selected parameters are as follows: preoperative analysis showed that the software output indicated a left palpebral fissure height of 10.23 mm vs. 12.70 mm on the right, a left MRD1 of 5.18 mm vs. 6.52 mm on the right, and a left brow-eye distance of 28.0 mm vs. 18.7 mm on the right. Postoperative analysis showed that left palpebral fissure height increased to 12.50 mm, left MRD1 increased to 6.40 mm, and left brow-eye distance decreased to 19.5 mm. These values are presented for illustrative purposes and rely on the common 11.77 mm iris diameter calibration; absolute physical accuracy has not been independently validated. Measurements for all seven parameters are presented in Table 3. The above represents the measurement output of the software on a single example image (Figure 5). For systematic evaluation of software performance, please refer to the preceding Sections 3.1–3.3.

**Table 3:** Preoperative and postoperative measurements of the illustrative case.

Parameter	Preoperative left	Postoperative left	Preoperative eye
Palpebral fissure height	10.23 mm	12.50 mm	12.70 mm
MRD1	5.18 mm	6.40 mm	6.52 mm
Brow-eye distance	28.0 mm	19.5 mm	18.7 mm
Brow height	38.2 mm	32.0 mm	31.4 mm
Medial canthal angle	53.66°	58.00°	58.74°
Lateral canthal angle	38.71°	46.00°	47.00°
Brow curvature	0.0164	0.0130	0.0124

## 4 Discussion

### 4.1 Strengths

In this preliminary single-center validation study, we developed and validated VisionMetric Suite, an AI-based automated periocular measurement tool. The software demonstrated high recognition efficacy, rapid analysis capability, and good agreement with both manual measurements and its own repeated measurements. User satisfaction was high (4.3/5.0), and all participating physicians expressed willingness to integrate the tool into routine clinical practice. As detailed in Section 3.2 and Table 1, satisfaction scores were similar across the three experience levels. Given the very small subgroup sizes, these descriptive findings should be viewed as preliminary usability observations rather than as statistical evidence of group equivalence. Junior residents mastered basic operations within 15 min, representing a significantly shortened learning curve compared to traditional measurement software, such as ImageJ which requires approximately 6 h of training (4), potentially enhancing workflow efficiency for ophthalmic plastic surgeons.

These findings are consistent with recent studies on AI-based ophthalmic measurement tools. The processing time of 2.8 s per image in our study is substantially faster than manual measurements, representing an efficiency improvement of over 98%, which aligns with the efficiency advantage reported by Park et al. (4). Rousseau and Retrouvey also confirmed that AI-based automated facial analysis significantly improves efficiency compared to manual measurements (21). Similarly, our mean ICC of 0.94 demonstrates comparable agreement. The 5.3% recognition failure rate, which may be attributed to image occlusion, low resolution, and facial deformation (Supplementary Figure S1), is consistent with the findings of Zhu et al. (22), highlighting the importance of standardized photographic protocols in clinical applications.

The reliability of VisionMetric Suite was further supported by its excellent agreement and repeatability ICC values. In this study, the two-way random effects model (ICC 2,1) was chosen because the two surgeons were considered random samples from a larger population of potential users, allowing generalization beyond these specific raters (23). The slightly higher repeatability ICC values compared to the agreement ICC values for most parameters reflect the inherent variability between human manual measurements and automated AI analysis, a pattern consistent with previous validation studies of AI-based measurement tools (24). The excellent repeatability (ICC = 0.92–0.99) confirms the stability of VisionMetric Suite across different users, indicating that the tool produces consistent and reproducible measurements even when operated by different individuals (19). These findings were corroborated by Bland–Altman analysis, which demonstrated narrow 95% limits of agreement and biases close to zero for all parameters, further supporting the strong agreement between AI and manual measurements under the same calibration assumption, as well as the high repeatability of the AI tool. Notably, while the AI tool demonstrated excellent repeatability for brow curvature (RAI ICC = 0.92, 95% CI: 0.87–0.96), its agreement with manual measurements for this composite parameter was slightly lower (AAM ICC = 0.87, 95% CI: 0.80–0.93), reflecting the inherent challenge of achieving perfect AI-manual alignment for complex morphological features.

Our validation of the software focused on seven core parameters: palpebral fissure height, MRD1, brow-eye distance, brow height, medial canthal angle, lateral canthal angle, and brow curvature (20). These parameters were selected because they exhibit the most significant and sensitive changes in congenital ptosis, the condition under investigation, and they span all four major output categories of VisionMetric Suite (eyelid morphology, brow morphology, brow-eye relationships, and angular measurements). Second, manually validating all 60 parameters is impractical, as each parameter requires precise landmark identification and measurement, a labor-intensive process that is time-consuming and prone to observer variability (25). Therefore, the current work should be viewed as a preliminary validation of a clinically important subset of parameters rather than a comprehensive validation of the full 60-parameter output space. The good agreement observed for these seven core parameters suggests that the remaining 53 parameters, which are derived from similar geometric relationships, may also be reliable, but this remains to be confirmed in future studies. We acknowledge that some unvalidated outputs may be composite or derived measures with error properties that differ from those of the validated subset, and further validation of additional parameters is needed for other clinical applications.

Recently, several AI-based tools have been developed for automated periocular measurement, including OrbitMap, PeriOrbitAI, DeepAAM, and the models reported by Nam et al. and Shao et al. OrbitMap, a fully automated deep learning-based tool, measures 38 parameters with a processing time of 1.45 s per image and reported ICC values ranging from >0.5 to 0.8 (8). While OrbitMap demonstrates acceptable efficiency, its agreement with manual measurements remains suboptimal for certain parameters. PeriOrbitAI, another fully automated open-source tool, processes eight parameters in 10.71 s per image but exhibits poor reliability, with ICC values below 0.5 for most measurements (8). DeepAAM focuses on four eyelid parameters (MRD1, MRD2, MIA1, MIA2) and was validated using *F*-test comparisons (*P* > 0.05) rather than ICC, limiting direct comparability (26). Nam et al. and Shao et al. reported automated systems for eyelid measurement, but both are limited to 3–4 parameters and lack comprehensive validation data (14, 15). In contrast, VisionMetric Suite offers several distinct advantages. First, with 60 parameters spanning eyelid morphology, brow morphology, brow-eye relationships, and interocular relationships, it provides the most comprehensive automated periocular assessment reported to date. Second, our mean ICC of 0.94 (range 0.87–0.98) demonstrates excellent agreement with manual measurements, substantially outperforming PeriOrbitAI (ICC < 0.5) and comparing favorably with OrbitMap (ICC > 0.5–0.8) (8). Third, the processing time of 2.8 s per image is faster than PeriOrbitAI (10.71 s) and significantly faster than semi-automated methods, while remaining competitive with the fastest reported tool (OrbitMap, 1.45 s) (8). Fourth, unlike most existing tools that are either not publicly available or lack open-source access, VisionMetric Suite is available upon request to facilitate further research and validation. Collectively, these preliminary findings suggest that VisionMetric Suite has the potential to be a comprehensive and efficient tool for periocular measurement in ophthalmic plastic surgery. However, given the single-center nature of this study and the limited sample size, larger multi-center studies are needed to confirm its reliability and generalizability.

The 60 measurement parameters cover a broad range of perioperative assessment needs in ophthalmic plastic surgery. Beyond the ptosis case presented in this report, the software may be also applicable to various periocular procedures, including blepharoplasty, medial canthoplasty, dermatochalasis correction, periorbital deformity correction, and periorbital injections. This is suggested by in our preliminary analysis, but further validation is required.

### 4.2 Limitations

Several limitations should be acknowledged. First, software accuracy depends on image quality: photographs require uniform illumination, clarity, neutral expression, absence of facial deformities, and unobstructed facial regions. This is reflected in the 5.3% recognition failure rate, which was primarily attributable to image clarity, facial occlusion, periocular deformities, and extreme head positions (Supplementary Table S3). Second, the current version is optimized for frontal views; oblique or lateral photographs may yield less reliable measurements. Third, while the iris diameter calibration method is widely used, individual variations in true iris diameter (typically 11.0–12.5 mm) introduce a potential systematic error of approximately ±5% (27). Given this inherent uncertainty, small differences should be interpreted as within the expected measurement variability rather than as highly precise absolute values. However, because the same fixed diameter was used for both AI and manual measurements, the comparisons between methods remain valid. Fourth, in patients with severe periocular deformities, automated landmark detection may require manual correction. Fifth, the software interface is currently available only in Chinese, which may limit its usability for non-Chinese-speaking users. Future versions will include multilingual support. Additionally, this study validated only seven out of 60 parameters. While these seven core parameters span all four major output categories and are clinically relevant to congenital ptosis, the reliability of the remaining 53 parameters has not been directly tested. Some unvalidated outputs may be composite or derived measures with error properties that differ from those of the validated subset. Therefore, the current findings should be interpreted as preliminary validation of a selected subset of clinically important parameters, and further validation of additional parameters is needed for other clinical applications. Finally, as a preliminary single-center validation study, the findings of this research are inherently limited in their generalizability. All data were collected from a single institution in China, and the patient population was exclusively of East Asian ethnicity. Therefore, the results may not be directly transferable to other populations, clinical settings, or imaging protocols. Multi-center studies involving diverse ethnic groups and varied clinical environments are essential to establish the external validity of VisionMetric Suite before it can be recommended for widespread clinical use.

### 4.3 Future directions

Future developments will focus on addressing the current limitations of the tool. Specific directions include integrating three-dimensional analysis to overcome the information loss inherent in two-dimensional imaging, introducing dynamic assessments such as blinking and gaze to capture functional states that static measurements cannot reflect, and establishing an online platform for multi-center data aggregation. These improvements will provide more comprehensive evidence-based support for perioperative evaluation in ophthalmic plastic surgery, facilitate physician-patient communication through the creation of intuitive visual models, and ultimately build an ophthalmic plastic surgery data platform to support high-quality clinical research. Furthermore, addressing the limitations of current samples and parameters, subsequent studies will extend validation to additional clinical parameters such as blepharoptosis and dermatochalasis, and evaluate the tool's applicability and generalizability across diverse ethnic populations and clinical settings, thereby enhancing its universality and clinical utility.

## 5 Conclusions

VisionMetric Suite integrates mature open-source machine learning frameworks with specialized periocular aesthetic morphology measurement algorithms, showing promise as an efficient, objective evaluation tool for preoperative assessment and postoperative outcome evaluation in ophthalmic plastic surgery. The software demonstrates strong agreement with manual measurements, fast turnaround time, and high user satisfaction within the limits of this single-center study. However, given the preliminary nature of this validation, these findings should be interpreted with caution. Multi-center studies with larger and more diverse populations are essential to establish the tool's generalizability and clinical utility before it can be recommended for widespread use.

## Data Availability

The raw data supporting the conclusions of this article will be made available by the authors, without undue reservation.
