# Supplementary material for: VisionMetric Suite for periocular measurement in ophthalmic plastic surgery: a preliminary single-center validation study
Source: Front Med (Lausanne). 2026 Jun 15;13:1855884. doi: 10.3389/fmed.2026.1855884 (PMC13310729; doi:10.3389/fmed.2026.1855884)
Supplement: Supplementary file 1 [file Supplementary_file_1.docx]

## Supplementary Table 1. Complete list of 60 periocular morphological parameters measured by VisionMetric Suite

| No. | Measurement Item | Description | Category |
| --- | --- | --- | --- |
| 1 | Left lateral canthal angle | Lateral canthal angle | Eye angles |
| 2 | Left medial canthal angle | Medial canthal angle | Eye angles |
| 3 | Left eye horizontal tilt angle | Eye horizontal tilt angle | Eye angles |
| 4 | jj distance | Intercanthal distance (medial) | Horizontal distance |
| 5 | oo distance | Interpupillary distance | Horizontal distance |
| 6 | ii distance | Intercanthal distance (lateral) | Horizontal distance |
| 7 | Left ij distance | Palpebral fissure length | Horizontal distance |
| 8 | Left oj distance | Medial canthus to pupil distance | Horizontal distance |
| 9 | Left od distance | MRD1 (marginal reflex distance 1) | Vertical distance (central) |
| 10 | Left oe distance | MRD2 (marginal reflex distance 2) | Vertical distance (central) |
| 11 | Left oa distance | Central pupil to brow upper margin distance | Vertical distance (central) |
| 12 | Left ob distance | Central pupil to brow lower margin distance | Vertical distance (central) |
| 13 | Left de distance | Central palpebral fissure height | Vertical distance (central) |
| 14 | Left dc distance | Central supratarsal fold height | Vertical distance (central) |
| 15 | Left cb distance | Central supratarsal fold to brow lower margin distance | Vertical distance (central) |
| 16 | Left ih distance | Lateral supratarsal fold height | Vertical distance (lateral) |
| 17 | Left ig distance | Lateral canthus to brow lower margin distance | Vertical distance (lateral) |
| 18 | Left if distance | Lateral canthus to brow upper margin distance | Vertical distance (lateral) |
| 19 | Left hg distance | Lateral supratarsal fold to brow lower margin distance | Vertical distance (lateral) |
| 20 | Left kl distance | Medial supratarsal fold height | Vertical distance (medial) |
| 21 | Left km distance | Medial lid margin to brow lower margin distance | Vertical distance (medial) |
| 22 | Left kn distance | Medial lid margin to brow upper margin distance | Vertical distance (medial) |
| 23 | Left kp distance | Medial palpebral fissure height | Vertical distance (medial) |
| 24 | Left eyelid fold area | Area enclosed by supratarsal fold and lid margin | Area measurement |
| 25 | Left L | Brow chord length (brow head to brow tail oblique distance) | Brow curvature |
| 26 | Left H | Brow chord height (brow peak to chord vertical distance) | Brow curvature |
| 27 | Left brow curvature | Brow curvature | Brow curvature |
| 28 | Left brow area | Brow area | Brow curvature |
| 29 | Left rtk angle | Brow curvature angle (brow head-brow peak-brow tail) | Brow curvature |
| 30 | Left ri angle | Canthal-brow angle (brow peak-lateral canthus horizontal angle) | Brow curvature |
| 31 | Left iris diameter L1 | Iris diameter | Scleral and iris data |
| 32 | Left iris area S1 | Iris area | Scleral and iris data |
| 33 | Left iris exposure area S2 | Corneal exposure area | Scleral and iris data |
| 34 | Left total eye area Ss | Total eye area | Scleral and iris data |
| 35 | Left scleral area S3 | Scleral area | Scleral and iris data |
| 36 | Left rk distance | Brow head to brow tail horizontal distance | Brow horizontal distances |
| 37 | Left rq distance | Brow tail to brow point 2 horizontal distance | Brow horizontal distances |
| 38 | Left ra distance | Brow tail to brow point 3 horizontal distance | Brow horizontal distances |
| 39 | Left rf distance | Brow tail to brow peak horizontal distance | Brow horizontal distances |
| 40 | Left fa distance | Brow peak to brow point 3 horizontal distance | Brow horizontal distances |
| 41 | Left fq distance | Brow peak to brow point 2 horizontal distance | Brow horizontal distances |
| 42 | Left aq distance | Brow point 2 to brow point 3 horizontal distance | Brow horizontal distances |
| 43 | Left or vertical | Brow tail to pupil vertical distance | Brow to pupil vertical distances |
| 44 | Left of vertical | Brow peak to pupil vertical distance | Brow to pupil vertical distances |
| 45 | Left oa vertical | Brow point 3 to pupil vertical distance | Brow to pupil vertical distances |
| 46 | Left oq vertical | Brow point 2 to pupil vertical distance | Brow to pupil vertical distances |
| 47 | Left ok vertical | Brow head to pupil vertical distance | Brow to pupil vertical distances |
| 48 | Left or oblique | Brow tail to pupil oblique distance | Brow to pupil oblique distances |
| 49 | Left of oblique | Brow peak to pupil oblique distance | Brow to pupil oblique distances |
| 50 | Left oa oblique | Brow point 3 to pupil oblique distance | Brow to pupil oblique distances |
| 51 | Left oq oblique | Brow point 2 to pupil oblique distance | Brow to pupil oblique distances |
| 52 | Left ok oblique | Brow head to pupil oblique distance | Brow to pupil oblique distances |
| 53 | Left rok angle | Brow tail-pupil-brow head angle | Brow to pupil angles |
| 54 | Left roq angle | Brow tail-pupil-brow point 2 angle | Brow to pupil angles |
| 55 | Left roa angle | Brow tail-pupil-brow point 3 angle | Brow to pupil angles |
| 56 | Left rof angle | Brow tail-pupil-brow peak angle | Brow to pupil angles |
| 57 | Left foa angle | Brow peak-pupil-brow point 3 angle | Brow to pupil angles |
| 58 | Left foq angle | Brow peak-pupil-brow point 2 angle | Brow to pupil angles |
| 59 | Left fok angle | Brow peak-pupil-brow head angle | Brow to pupil angles |
| 60 | Left aoq angle | Brow point 3-pupil-brow point 2 angle | Brow to pupil angles |

Note: All parameters are measured for both left and right eyes. The software generates bilateral measurements automatically.

**Supplementary Table 2.** Demographic characteristics of the image dataset (N = 226)

| Characteristic | Category | n | % |
| --- | --- | --- | --- |
| Age group | 18–30 years | 68 | 30.1 |
|  | 31–45 years | 82 | 36.3 |
|  | 46–60 years | 54 | 23.9 |
|  | >60 years | 22 | 9.7 |
| Sex | Male | 58 | 25.7 |
|  | Female | 128 | 56.6 |
|  | Unspecified | 40 | 17.7 |
| Diagnosis | Normal eyes | 80 | 35.4 |
|  | Ptosis | 26 | 11.5 |
|  | Post-blepharoplasty | 82 | 36.3 |
|  | Dermatochalasis | 28 | 12.4 |
|  | Other | 10 | 4.4 |
| Ethnicity | East Asian (Chinese) | 226 | 100 |

## Supplementary Table 3. Analysis of recognition failure cases (n = 12, total N = 226)

| Failure category | n (%) | Description |
| --- | --- | --- |
| Facial occlusion | 5 (41.7) | Hair strands, hats, or small foreign bodies partially obscuring the facial region |
| Poor image resolution / blurring | 3 (25.0) | Low image quality, motion blur, or inadequate focus reducing landmark contrast |
| Head pose variation | 2 (16.7) | Head rotation or tilt within the acceptable clinical range but still causing landmark projection error |
| Severe periocular deformity | 2 (16.7) | Severe ptosis with anatomical deviation from typical configuration |
| **Total** | **12 (100)** | — |

Supplementary Table 2 summarizes the 12 recognition failure cases (5.3% of 226 images) after applying the basic pre‑selection criteria. The most common cause was facial occlusion (n=5, 41.7%), followed by poor image resolution or blurring (n=3, 25.0%), head pose variation (n=2, 16.7%), and severe periocular deformity (n=2, 16.7%).


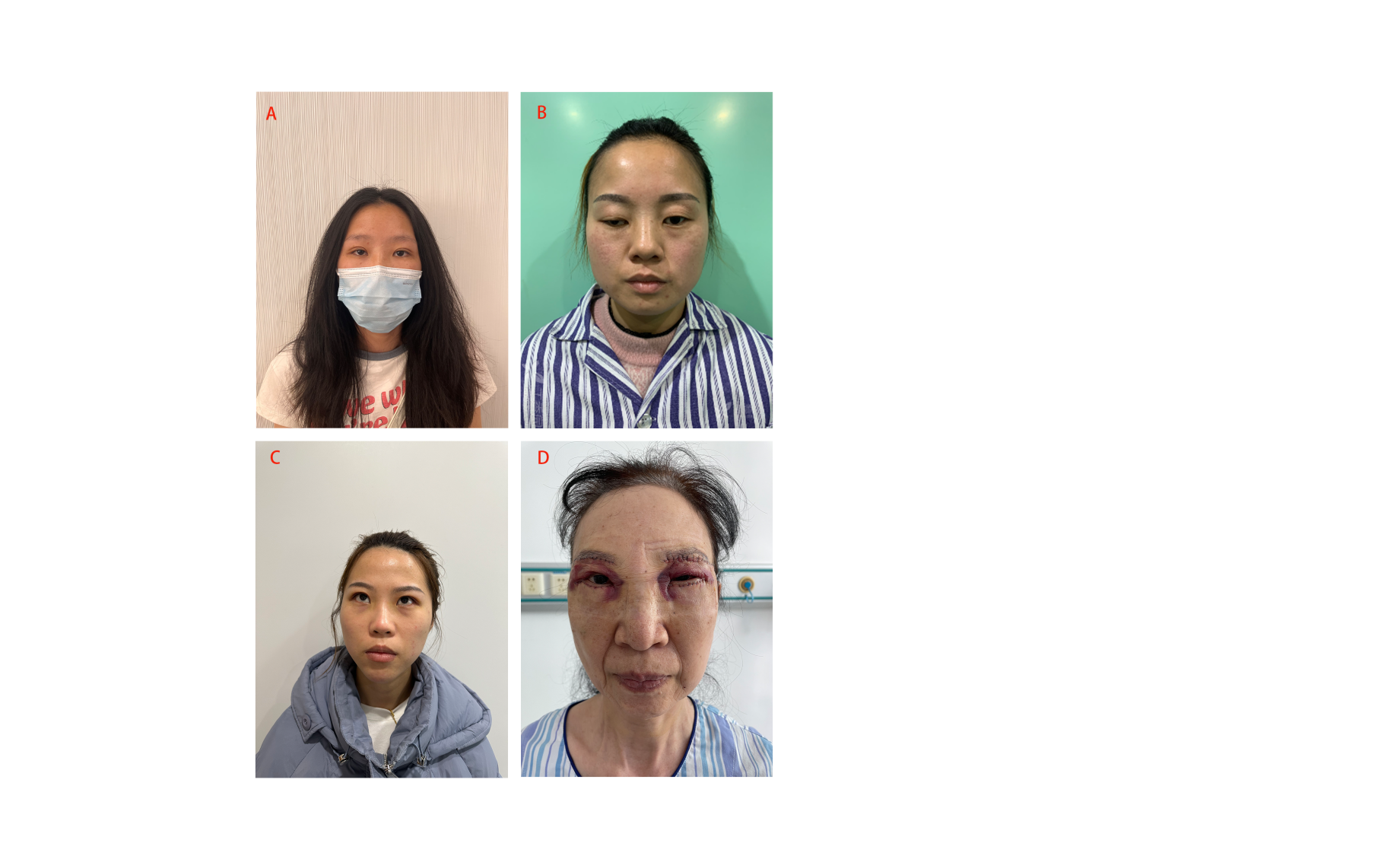


**Supplementary Figure S1. Representative examples of recognition failure cases corresponding to Supplementary Table 3.** (A) Facial occlusion: surgical mask partially covering the lower face. (B) Poor image resolution. (C) Inappropriate head posture. (D) Severe periocular deformity: severe postoperative swelling following brow lift combined with lower blepharoplasty, resulting in anatomical deviation from typical eyelid and brow configuration. These images represent the 5.3% recognition failure rate reported in Section 3.1.
